# Structural transition in the collective behavior of cognitive agents

**DOI:** 10.1038/s41598-019-48638-8

**Published:** 2019-08-28

**Authors:** Hannes Hornischer, Stephan Herminghaus, Marco G. Mazza

**Affiliations:** 1 Institute of Systems Sciences, Innovation and Sustainability Research, University of Graz, Merangasse 18/1, 8010 Graz, Austria; 2 Max Planck Institute for Dynamics and Self-Organization (MPIDS), Am Faßberg 17, 37077 Göttingen, Germany; 3 Interdisciplinary Centre for Mathematical Modelling and Department of Mathematical Sciences, Loughborough University, Loughborough, Leicestershire LE11 3TU United Kingdom

**Keywords:** Statistical physics, Statistical physics, thermodynamics and nonlinear dynamics

## Abstract

Living organisms process information to interact and adapt to their surroundings with the goal of finding food, mating, or averting hazards. The structure of their environment has profound repercussions through both selecting their internal architecture and also inducing adaptive responses to environmental cues and stimuli. Adaptive collective behavior underpinned by specialized optimization strategies is ubiquitous in the natural world. We develop a minimal model of agents that explore their environment by means of sampling trajectories. The spatial information stored in the sampling trajectories is our minimal definition of a cognitive map. We find that, as cognitive agents build and update their internal, cognitive representation of the causal structure of their environment, complex patterns emerge in the system, where the onset of pattern formation relates to the spatial overlap of cognitive maps. Exchange of information among the agents leads to an order-disorder transition. As a result of the spontaneous breaking of translational symmetry, a Goldstone mode emerges, which points at a collective mechanism of information transfer among cognitive organisms. These findings may be generally applicable to the design of decentralized, artificial-intelligence swarm systems.

## Introduction

The collective behavior of simple agents can exhibit a stunning degree of organization, as can be seen in the emergence of a mound from a cell colony of *Dictyostelium discoideum*, the construction of a complex termitarium by a termite colony, or the successful defense against predators by swarming starlings or a school of fish. Although the individual agents respond to environmental stimuli in a local, individual, and in most cases unconscious manner, the result often appears as beautifully orchestrated. Such phenomena naturally prompt the general question what are the features of such ‘intelligent’ collective behavior in a society of individuals with rather limited individual cognitive abilities.

It is therefore of great interest to investigate the impact of the cognitive competence of individual actors on their collective behavior from a fundamental point of view. This requires the definition of notions of cognition which are at the same time sufficiently general to resemble the wide class of agents encountered in collective phenomena, but at the same time are sufficiently simple to be accessible to statistical physics methodology. In search for such a minimal model, we observe that the navigation of agents through their environment requires a feedback loop of information processing, inference, and active response^1–3^. Survival requires living organisms to constantly incorporate information of their environment into some kind of internal representation of the exterior^4^. This internal representation can be as sophisticated as a human’s ability to navigate a complex metropolitan road network, or more rudimentary like the map-like spatial memory in the navigation of honey bees^5–9^.

A cognitive agent must be able to predict future events^1^. Picture a gazelle trying to evade a pursuing lion. Drawing on its knowledge of its immediate surroundings, the prey will choose a path leading to, for example, open space with multiple possibilities of escape, rather than a path leading to a cul-de-sac. As another example —particularly accessible to formal modeling— we may consider a chess player, whose activities take place in the abstract space of moves on the chessboard. It is the player’s internal cognitive map that allows her to contemplate the possible moves and the ramifications of their consequences. Depending on the experience and skills of the player, she will be able to contemplate her possible next move, her opponent’s countermove, and possibly additional moves in response. The player, in other words, will sample hypothetical sequences of steps (i.e. trajectories) in the abstract space of moves within the chessboard.

The player’s strategy can be cast into a simple, general form: the maximization of future options to move without losing the king. The number of moves the player is able to contemplate ahead is a direct numerical measure of her cognitive competence. In the present study, we adopt a straightforward generalization of this measure by defining cognitive competence as the ability of an agent to determine the number of possible moves within a given environment. This ability depends upon the agent’s cognitive map, and we may assume that, similarly to the chess player, the agent will seek to maximize its number of future options to move.

We will now describe a mechanistic implementation of the above ideas which has the twofold advantage to (*i*) make our ideas concrete, and (*ii*) provide a connection to the field of active matter^10,11^. We consider a set of freely mobile, identical, spherical particles with diameter *σ*. Their only ‘cognitive’ activity is to explore the surrounding space. This exploration is performed via *hypothetical* random walks of a certain length, starting from the agent’s current position, and by evaluating the shape of such walks they gain knowledge about the location of other particles or confining boundaries. More precisely, each agent performs a fixed number *N*_*ω*_ of such walks and evaluates how elongated the hypothetical trajectory of this walk is, thus allowing the inference of the location of confining objects, where the trajectory is likely to be more compact, and empty areas, where the trajectory can be more elongated and spacious. As measure for quantifying the configuration of each hypothetical trajectory we use the radius of gyration (see Methods). Clearly, the larger the cognitive competence of the agent is (i.e. the longer these hypothetical walks can be made), the larger the cognitive map of the environment will be.

A formal analogy between random walks and ideal polymers^12,13^ can help us understand what to expect. If the random walks were really executed, with no crossings allowed, the resulting interaction would be analogous to the repulsion experienced between star-copolymer molecules with *n*_*ω*_ chains. The mutual interaction of polymer chains is known to be characterized by isotropically repulsive entropic forces^14^. We will demonstrate below that by replacing the real random walks with hypothetical, sampling walks, hence introducing cognitive maps into the system, the collective behavior changes dramatically.

For each agent, we consider a set of random, hypothetical sampling trajectories, {Γ_*τ*_(*t*)}, each of total duration *τ*, that the agent may traverse to explore its environment. Following an earlier work where these hypothetical walks have been introduced^15^, we explicitly indicate the dependence on time *t* because the cognitive map is dynamically updated as information is acquired. Starting from its initial position, **r**_0_, the region probed by the agent, and thus the size of its cognitive map, is directly proportional to *τ*.

We can build a probabilistic description of this cognitive map by considering the probability density function *P*(Γ_*τ*_(*t*)|**r**_0_) associated to an ensemble of trajectories all starting from **r**_0_. Our probabilistic description should however represent mathematically the information acquired in building a cognitive map. According to Shannon^16,17^, −*P*ln*P* is the most general functional form that obeys the constraints of continuity, nonnegativity, and additivity of information. It is then natural to express the information content stored in the cognitive map as

1 $${\mathscr{S}}({\bf{r}};\tau )=-\,{k}_{{\rm{B}}}\int \,P({{\rm{\Gamma }}}_{\tau }(t)|{\bf{r}})\mathrm{ln}\,P({{\rm{\Gamma }}}_{\tau }(t)|{\bf{r}})\,{\mathscr{D}}{{\rm{\Gamma }}}_{\tau }(t),$$

which is a path integral over the hypothetical trajectories of an agent at position **r**, building up the cognitive map {Γ_*τ*_(*t*)} (see Methods and ref. ^15,18^), and where *k*_B_ is Boltzmann’s constant to give dimensions of entropy.

The central assumption of the present work shall be that intelligent agents tend to maximize the information content stored in their cognitive maps. This assumption, together with Eq. (1), immediately implies that a cognitive agent tends to maximize the diversity of possible future trajectories. As a cognitive agent acquires information about the external environment, which is by nature of the process limited and partial, the most *unbiased* decision the agent can make is the one corresponding to the maximum of entropy, because it uses all the available information without any additional assumptions. Mathematically, it assigns a positive weight to every situation which is not excluded by the given information^19,20^.

Maximization of ${\mathscr{S}}$ can then be represented by a force acting on the agent of the form

2 $${\bf{F}}({\bf{r}};\tau )=\theta \nabla {\mathscr{S}}({\bf{r}};\tau ),$$

where the coupling parameter *θ* (with dimensions of temperature) quantifies the cognitive competence of the agent, that is, how strongly the agent responds to the environment (see Methods). In order to maximize the information content of the cognitive map, an agent will move by following the gradient of ${\mathscr{S}}$.

An intuitive understanding of the principle stated above can be gained by considering again the predator-prey system discussed above. The prey will choose a path which maximizes the future available options (and hence its survival probability). More formally, the approach presented here is indebted to a number of contributions in complex system theory. The well-informed reader will recognize echoes of Kauffman’s hypothesized ‘fourth law of thermodynamics’^21^, stating that autonomous agents maximize the average secular construction of diversity^21^, e.g. organisms tend to increase the diversity of their organization. In the context of information-processing systems, related approaches have been expressed by Linsker^22^ with his “Infomax” principle which was used to demonstrate the emergence of structure in models of neural architecture^23–25^, or Ay *et al*.^26^ with the maximum of a predictive information —a relation between future states and past ones— biological infotaxis^2^, sensorimotor systems^27^, and control theory^15^. Our approach is based on the idea that cognitive systems entail some mechanism of prediction^28,29^. We therefore consider a finite duration *τ* of the hypothetical trajectories.

The motivation for our approach is that an optimal information-processing dynamics should indicate the level of competence of the agent to respond to complex stresses and stimuli. To name only a few examples where maximization of information or entropy has been found empirically and might constitute a fundamental mechanism, maximization of information has been measured as a characteristic of human cognition^30,31^; a pair-wise maximum entropy accurately models resting-state human brain activity^32^; patients with ADHD exhibit reduced signal entropy as compared with healthy individuals^33^.

**Figure 1:**
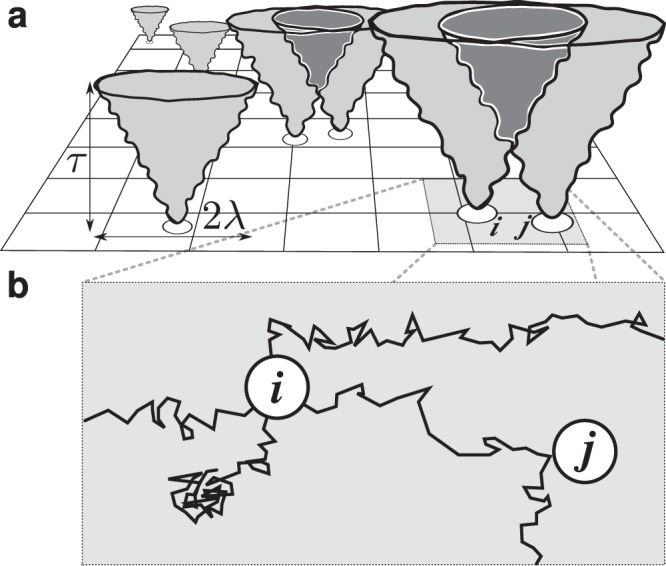
Schematic representation of a system of cognitive agents and their cognitive maps. (**a**) Starting from the initial condition in configuration space the agents (empty circles) create a cognitive map of their surroundings by means of hypothetical sampling trajectories of duration *τ*. The cones show a (2 + 1)-dimensional envelope of the trajectories, which represent the cognitive maps, of average radius *λ*. When the cones overlap, the agents sense each other and a force arises. (**b**) Shown here are four sampling trajectories emanating from cognitive agent *i*. As one of the trajectories impacts with agent *j*, agent *i* is forced to modify its trajectory, thus responding to its cognitive representation of the environment. Cognitive competence is the tendency to maximize the options left after one agent enters the space of the other, and to avoid in the most efficient way the overlapping regions.

Figure 1 shows a schematic of a few agents moving on a two-dimensional space, whereas the vertical dimension represents time. Agents interact with each other and with the environment. Each agent explores the available configuration space and acquires information about its structure, and in so doing builds its cognitive map, and optimizes its behavior through responding to the surrounding. The hypothetical trajectories, exploring the available space, have an envelope characterized by a spatial extension *λ*, and temporal extension *τ*. In the overlap regions of the forward cones the agents have a probability to collide. This possibility gives rise to the effective force **F**(**r**; *τ*). The overlap regions and the corresponding effective forces appear when the distance between any two agents becomes shorter than the average linear length of the agents’ hypothetical trajectories, which relates to the size of the cognitive map.

Our definition of information entropy ${\mathscr{S}}$ satisfies the following criteria. First, ${\mathscr{S}}$ is based on the information content of the system because agents retrieve and process information about the presence of other agents. Second, it does not require any specific goal or strategy, such as rules for taxis of bacteria in chemo-attractant concentration fields. Third, it obeys the laws of information theory and information processing, essential to build cognitive maps. Fourth, it obeys causality because the current state of the cognitive map influences the agent’s future dynamics.

## Results

We carried out simulations of *N* identical agents in a two-dimensional, continuous system of size *L*×*L*, where agents interact with each other via the cognitive force **F** and via hard-core repulsion when their distance is less than the agent’s diameter *σ*. The agents’ configurations evolve continuously from a random initial distribution towards steady-state configurations for different sizes *λ* of the cognitive map. We define the size of a cognitive map as the average distance between start and end of hypothetical trajectories $\lambda =\frac{1}{{N}_{{\rm{\Omega }}}}{\sum }_{n=1}^{{N}_{{\rm{\Omega }}}}\,|{{\bf{r}}}_{{{\rm{\Gamma }}}_{n}}(\tau )-{{\bf{r}}}_{{{\rm{\Gamma }}}_{n}}(0)|$, where *N*_Ω_ is the total number of hypothetical sampling trajectories.

**Figure 2:**
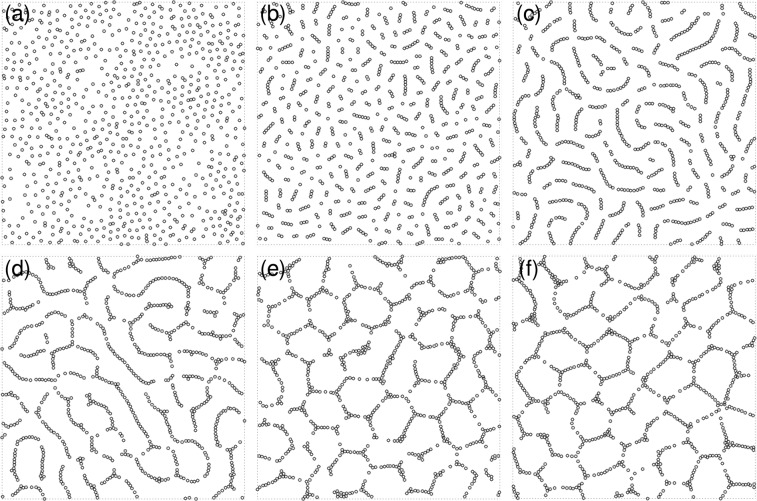
Steady-state configurations of a cognitive-agent system. Snapshots of a two-dimensional system with filling fraction *ϕ* = 0.1 (*N* = 800, *L* = 80*σ*) in steady-state configurations. As the size of the cognitive map *λ* increases, complex structures emerge. (**a**) At *λ* = 0.8*σ* the agents are randomly distributed. (**b**) At *λ* = 3.2*σ* agents start forming short linear chains. (**c**) At *λ* = 5.6*σ* the chains outline a labyrinthine pattern. (**d**) At *λ* = 7.6*σ* the labyrinthine pattern continuously changes into a cellular structure. At *λ* = 10*σ* (**e**) and *λ* = 10.8*σ* (**f**) the cellular structure is well formed. Thus, cognitive agents capable of ‘intelligent’ response to their environment and maximizing their cognitive map information exhibit a clear phase transition from disorder (at small *λ*) to order (at large *λ*).

Figure 2 shows the steady-state configurations of the system as the size *λ* of the map increases. At low values of cognitive map size *λ* with respect to the inter-agent separation, most agents are isolated and randomly distributed throughout the system (Fig. 2a). The agents try to stay as far apart as possible from each other in an attempt to maximize their available space. For a horizon of linear size *λ*, the available space scales as *λ*^2^. As *λ* increases, we observe the spontaneous formation of short linear chains of agents (Fig. 2b). At *λ* = 5.6*σ*, the chains grow longer and outline a labyrinthine pattern in the system (Fig. 2c). The emergence of this spatial organization can be understood as a way to increase the available configuration space in the direction normal to the chain-like structures. Consider chains of typical length $\ell$. Once chains are formed, the space available to their horizons scales approximately as $\ell \lambda$. Thus, the ratio of available space between chains and the disordered configuration scales approximately as $\ell /\lambda$. The agents can therefore increase their available space by increasing $\ell$, and forming long chains.

Upon further increase of *λ*, the pattern continuously turns into a cellular structure (Fig. 2d,e), which we find well developed at *λ* = 10.8*σ* (Fig. 2f). Consider now the (entropically) advantageous strategy of agents to form chains (e.g. for *λ* = 5.6*σ*). With increasing *λ* agents tend to arrange such that they keep larger distances from other chains of agents within proximity. Due to the fixed filling fraction and size of the system this leads to chains connecting to joints and ultimately cellular structures, of roughly hexagonal symmetry, which provides the optimal tiling of the plane.

**Figure 3:**
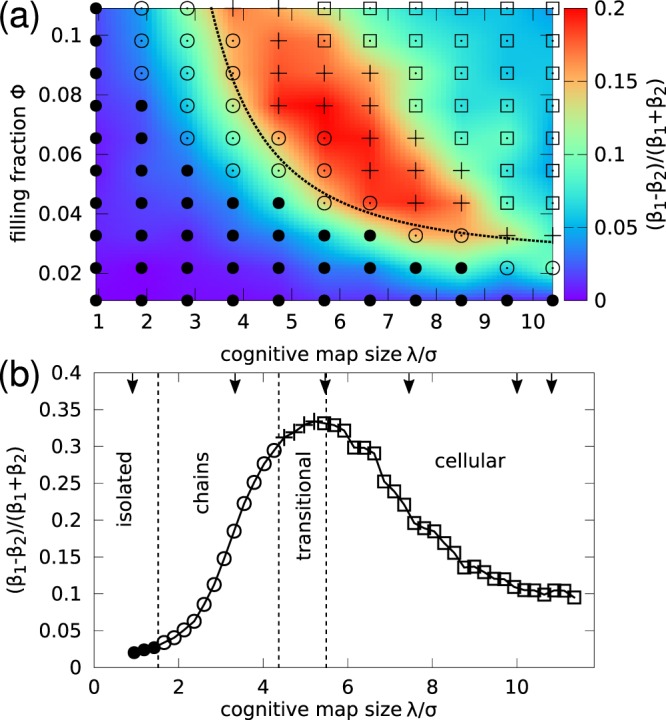
Nonequilibrium phase diagram of the cognitive agent system. (**a**) Symbols represent the steady-state configuration for up to *N* = 500 agents in a system of size *L* = 60*σ*: isolated agents (●), short chains (⚬), labyrinthine pattern (+), cellular pattern (□). Heat map of the anisotropy $\alpha \equiv \frac{{\beta }_{1}-{\beta }_{2}}{{\beta }_{1}+{\beta }_{2}}$ of the ${W}_{2}^{\mathrm{1,1}}$ Minkowski tensor. The dotted line represents the *ϕ*^−1/2^ scaling of the transition values of *λ* (note that an offset is added because at very low filling fraction no transition is expected as there is now significant overlap of the cognitive maps). (**b**) Dependence of the anisotropy $\alpha \equiv \frac{{\beta }_{1}-{\beta }_{2}}{{\beta }_{1}+{\beta }_{2}}$ on the cognitive map size *λ* for a system at *ϕ* = 0.1 (*N* = 800, *L* = 80*σ*). Symbols have the same meaning as in panel (a). Cognitive agents exhibit an order-disorder phase transition to a cellular pattern as the mean inter-agent distance becomes comparable to the cognitive map size *λ*. The arrows indicate the values of *λ* for the configurations shown in Fig. 2.

A similar sequence of patterns can be observed when we vary the filling fraction, *ϕ* ≡ *Nπσ*^2^/(4*L*^2^). The phase diagram of the system is shown in Fig. 3a. The transition line from short chains to more complex patterns is well fitted by a relation $\varphi  \sim {\lambda }^{-2}$ which suggests that the transition is triggered as the mean inter-agent distance becomes comparable to the cognitive map size *λ*.

In order to analyze the complex morphology of the patterns, we employ the anisotropy parameter $\alpha \equiv \frac{{\beta }_{1}-{\beta }_{2}}{{\beta }_{1}+{\beta }_{2}}$, defined in terms of the eigenvalues *β*_1_ and *β*_2_ of the Minkowski tensor^34,35^, which is a measure of anisotropy of configurations of particles (see Methods for more details). Figure 3a shows as a heat map the association of the phase diagram with the anisotropy *α* of the configurational patterns. At fixed filling fraction *ϕ*, the system exhibits the largest anisotropy *α* when linear chains start to connect with each other for intermediate values of the size *λ* of the cognitive map. In contrast, at low *λ*, where agents are isolated, the system is trivially isotropic. At large values of *λ*, where cognitive maps significantly overlap and cellular patterns emerge (Fig. 2f), the associated anisotropy decreases to values that are however larger than in the case of low *λ*. This indicates that the system gains again isotropy on the larger scale of the cells. Figure 3b shows the anisotropy *α* of the pattern. It exhibits a sharp maximum at $\lambda \simeq 5.5\sigma$ where the linear chains are most pronounced and the system is at the threshold of forming the labyrinthine patterns. The transition from isolated particles to cellular structures appears to be continuous, as no structural or dynamical observable shows discontinuous behavior. The transition occurs when *α* is considerably larger than zero, that is, for $\lambda \simeq 2\sigma$.

Our results so far illustrate that as the cognitive maps of the agents overlap, interesting collective behavior emerges. A moment’s reflection will show that agents perceive each other’s presence via their respective cognitive maps, and thus information about each other’s presence must be exchanged, and this information flow, in turn, dynamically modifies the cognitive maps of the agents. Quantifying the information flow among agents will instruct us on the origin of the collective behavior.

This information flow can be quantified via the notion of mutual information^36–42^. The mutual information for two random variables *a* and *b* is given by ${\sum }_{a,b}\,P(a,b){\mathrm{log}}_{2}\frac{P(a,b)}{P(a)P(b)}$, where *P*(*a*) represents the probability distribution of *a*, and *P*(*a*, *b*) is the joint probability distribution. Because we are interested in isolating the causal interaction between agents that underpins the update of the cognitive maps, we consider the positions (*x*_*i*_(*t*), *y*_*i*_(*t*)) of the *i*-th agent at time *t* and compute ${\overrightarrow{M}}_{ij}\equiv ({M}_{ij}^{x},{M}_{ij}^{y})$, between agent *i* and *j*, where ${M}_{ij}^{x}=P({x}_{i},{x}_{j}){\rm{l}}{\rm{o}}{{\rm{g}}}_{2}\frac{P({x}_{i},{x}_{j})}{P({x}_{i})P({x}_{j})}$, and ${M}_{ij}^{y}=P({y}_{i},{y}_{j}){\mathrm{log}}_{2}\frac{P({y}_{i},{y}_{j})}{P({y}_{i})P({y}_{j})}$. The total mutual information is then

3 $$ {\mathcal M} =\frac{1}{{N}_{p}}\sum _{\langle i,j\rangle }\,|{\overrightarrow{M}}_{ij}|,$$

where 〈*i*, *j*〉 represent all pairs of agents which are within a local neighborhood of distance 4*σ* (larger values of this cutoff do not qualitatively change the results), and *N*_*p*_ is the total number of neighbor pairs (*i*, *j*) included in the sum in Eq. (3).

**Figure 4:**
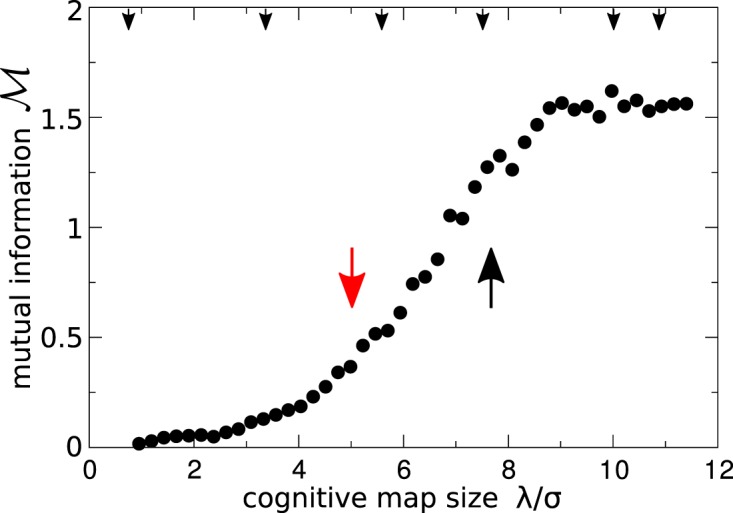
Mutual information as an order parameter in the transition of cognitive agents. As the size of the cognitive map *λ* increases, the mutual information ${\mathcal M}$ among the cognitive agents increases once their maps overlap significantly. The calculations of the mutual information ${\mathcal M}$ depending on *λ* were done for a system of size *L* = 80*σ* at fixed number density. The results are averaged over eight independent simulations for each data point. The small arrows on the top abscissa axis indicate the values of *λ* for the configurations shown in Fig. 2, whereas the large black and red arrows mark the location of the systems considered in Fig. 6.

Figure 4(a) shows the dependence of the mutual information ${\mathcal M}$ on the size of the cognitive map *λ*. At very small *λ*, the cognitive agent system exhibits a mutual information which is nearly vanishing on account of the nearly independent motion of each agent. As the size of the cognitive map increases beyond $\lambda \simeq 2.0\sigma$, ${\mathcal M}$ increases steadily, while the system develops labyrinthine and cellular patterns. At $\lambda \simeq 9.5\sigma$, ${\mathcal M}$ reaches a plateau value, corresponding to well-developed cellular patterns. These results show that upon increasing the size of the cognitive maps, an indirect exchange of information takes place among the agents, which in turn leads to the formation of complex structures.

The information exchange among the agents is correlated to their structural transition. To quantify the degree of correlated motion, we turn to a standard tool for the analysis of the dynamic response of many-body systems^43^: the displacement covariance matrix^44^ *C*_*ij*_ ≡ 〈*δ***r**_*i*_(*t*)⋅*δ***r**_*j*_(*t*)〉_*t*_, where *δ***r**_*i*_(*t*) ≡ **r**_*i*_(*t*) − 〈**r**_*i*_〉, and the angle brackets with subscript *t* indicate an average over time; the eigenvalues and eigenvectors of *C*_*ij*_ provide information about the coherent motion in the system. Provided that the particles have well-defined average positions 〈**r**_*i*_〉 —and this is in fact the case once complex patterns emerge— the spectral properties of *C*_*ij*_ can describe effective excitation modes^45^.

**Figure 5:**
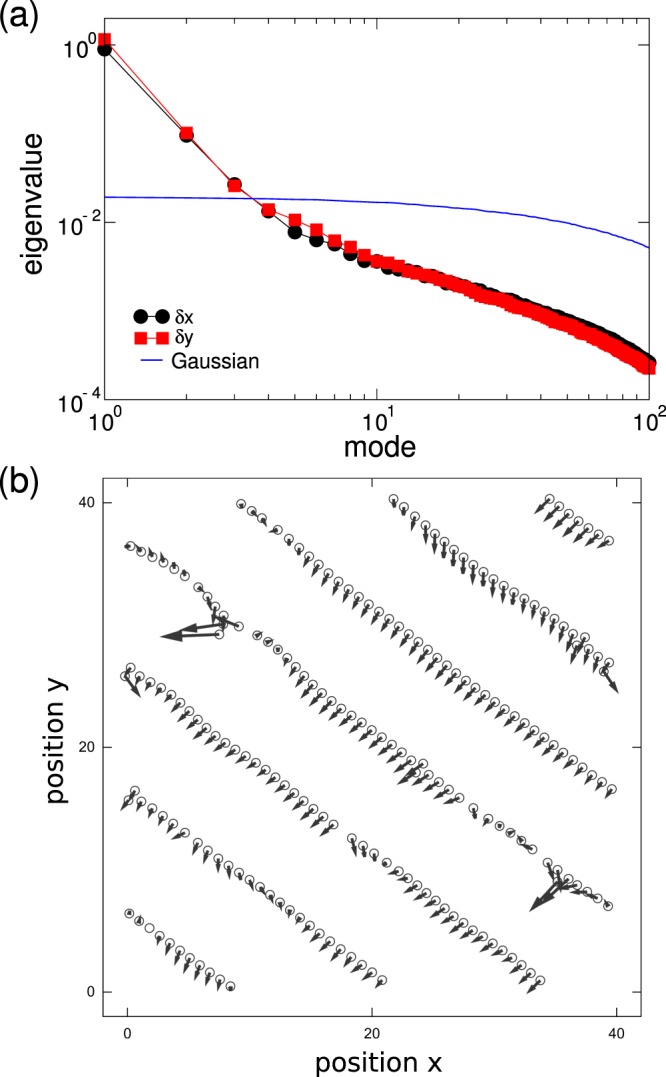
Growth of correlated motion. (**a**) Spectrum of eigenvalues of the displacement covariance matrix (for a system with *ϕ* = 0.1 (*N* = 200, *L* = 40*σ*) and *λ* = 8*σ*) and comparison with a model of Gaussian-distributed random displacements. The largest eigenvalue mode corresponds to a Goldstone mode propagating through the system. (**b**) We show the collective fluctuation in the agents’ positions due to a Goldstone mode propagating through the system when elongated linear chain patterns emerge. As complex patterns emerge and Galilean invariance is spontaneously broken, collective fluctuations materialize through the propagation of a Goldstone mode.

Figure 5a shows the eigenvalues of *C*_*ij*_ for *δx* and *δy*, and the comparison with the uncorrelated motion generated with a random matrix model of Gaussian-distributed displacements. The first mode of the cognitive system is considerably above the random Gaussian model, and corresponds to a large wavelength mode propagating through the system. This collective mode is shown in Fig. 5b. This is the Goldstone mode associated to the structural transition and corresponds to excitations of the ordered state. The situation is reminiscent of equilibrium systems where the spontaneous breaking of translational symmetry (Galilean invariance) is associated with the emergence a massless Goldstone mode, propagating through the system with a scale-free correlation length. Goldstone modes have been also identified in models and observations of active collective behavior^46–48^. In practice, this means that in certain configurations of the system some fluctuations propagate very quickly throughout the system, and they do not depend on the system size.

**Figure 6:**
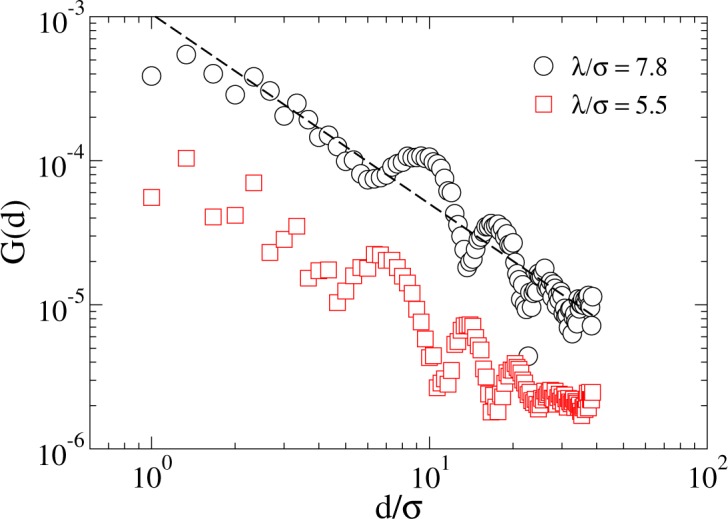
Spatial correlations. The correlation *G*(*d*) in the motion of *N* = 800 (*L* = 80*σ*, *ϕ* = 0.1) agents. *G*(*d*) exhibits a long-range nature, compatible with the presence of long-range excitation in a system with symmetry breaking. The oscillation superimposed to the decay of *G*(*d*) is due to the cellular pattern of the system.

Figure 6 shows the spatial correlation of displacements between agents defined via the correlation function *G*(*d*) = 〈*δ***r**_*i*_(*t*)⋅*δ***r**_*j*_(*t*)〉_*t*,(*i*,*j*)_, where *d* ≡ |**r**_*i*_(*t*) − **r**_*j*_(*t*)|, and the angle brackets with subscript *t*, (*i*, *j*) indicate an average over time and pairs of agents (*i*, *j*) separated by distance *d*. The correlation in the agents’ motion decays with distance with a power law (the oscillations with decaying amplitudes are due to the cellular pattern of the system).

In summary, our study furnishes a first step forward in the understanding of nonequilibrium transitions in a system of cognitive agents that dynamically interact with their environment, and respond to it with cognitive competence by maximizing the information content of their cognitive maps. The transition from isolated particles to complex patterns is characterized by different degree of overlap of the cognitive maps. The continuous change of ${\mathcal M}$ as the system develops complex patterns, together with the change of the anisotropy parameter $\frac{{\beta }_{1}-{\beta }_{2}}{{\beta }_{1}+{\beta }_{2}}$ point at a transition in cognitive-agent systems. We have identified a Goldstone mode propagating through the system that is generated by the spontaneous symmetry breaking of the structural transition as complex patterns emerge.

Apart for its significance for investigations of complex organisms whose active response to environmental stimuli is based on various levels of cognition, from eusocial insects to mammals, our results are relevant to artificial systems like autonomous micro-robots, and swarm robotics^49,50^, which are explicitly designed to autonomously mimic the collective behavior of living organisms.

## Methods

### Simulations

Every agent obeys the following equation of motion

4 $$m\dot{{\bf{v}}}=-\gamma {\bf{v}}+{\bf{F}}({\bf{r}};\tau )+{\bf{h}}({\bf{r}}),$$

where **v** is the velocity of the agent, *m* its mass, *γ* the viscous drag, **F**(**r**;*τ*) the cognitive force, and **h**(**r**) is the short-range repulsion among agents, modeled via a repulsive linear spring when |**r**_*i*_ − **r**_*j*_| < *σ*, where *σ* is the hard-core diameter of the agents.

As described above, the calculation of the cognitive force **F**(**r**;*τ*) [Eq. (2)] requires calculating a set of hypothetical sampling trajectories {Γ_*τ*_(*t*)}. The simulation algorithm is based on the following two steps: (*i*) generation of the hypothetical trajectories, resulting in the construction of the cognitive map, and computation of the cognitive force **F**(**r**;*τ*) (see below for details); (*ii*) update of the agent’s position according to Eq. (4). During the generation of the hypothetical trajectories, all agents in the system remain fixed in their current positions. The dynamics of the system evolve by repeating the two steps above.

The agents are initially placed randomly with a uniform distribution within the system and without any overlap of the agents’ hard cores. The system size is, unless otherwise specified, fixed at *L* = 80*σ*.

### Construction of the cognitive map

The calculation of *P*(Γ_*τ*_(*t*)|**r**_0_) is performed by generating hypothetical sampling trajectories, each of which represents a virtual evolution during the time [0,*τ*] of the agent with constraints fixed at the present configuration and not depending on time. The hypothetical trajectories are generated using Langevin dynamics

5 $$m\dot{{\bf{v}}}=-\gamma {\bf{v}}+\xi (t)+{\bf{h}}({\bf{r}}),$$

where **v**, *m*, *γ*, and **h(r)** have the same meaning as in Eq (4), *ξ*(*t*) is a random noise with zero mean and 〈*ξ*_*i*_(*t*)*ξ*_*j*_(*t*′)〉 = 2*γk*_B_*Tδ*_*ij*_*δ*(*t* − *t*′).

Any interaction of a hypothetical sampling trajectory with another agent is hard-core repulsive, that is the trajectory is reflected elastically by the other agent.

### Derivation of the cognitive force

Here we show the derivation of an expression for the entropic force we use to calculate the system’s dynamics. This is a derivation adapted and simplified from^15^. We start by using Eqs (1) and (2) and consider the gradient in (2) with respect to position space coordinates at present time **r**(*t* = 0) = **r**(0) and arrive at

6 $$\begin{array}{ccc}{\bf{F}}({\bf{r}}(0);\tau ) & = & -\theta {k}_{{\rm{B}}}\frac{{\rm{\partial }}}{{\rm{\partial }}{\bf{r}}(0)}\int \,Pr({{\rm{\Gamma }}}_{\tau }|{\bf{r}}(0))\,{\rm{l}}{\rm{n}}\,[Pr({{\rm{\Gamma }}}_{\tau }|{\bf{r}}(0))]{\mathscr{D}}{{\rm{\Gamma }}}_{\tau }\\  & = & -\theta {k}_{{\rm{B}}}\int \,\frac{{\rm{\partial }}Pr({{\rm{\Gamma }}}_{\tau }|{\bf{r}}(0))}{{\rm{\partial }}{\bf{r}}(0)}\,{\rm{l}}{\rm{n}}\,[Pr({{\rm{\Gamma }}}_{\tau }|{\bf{r}}(0))]\\  &  & +\,Pr({{\rm{\Gamma }}}_{\tau }|{\bf{r}}(0))\cdot [\frac{1}{Pr({{\rm{\Gamma }}}_{\tau }|{\bf{r}}(0))}\cdot \frac{{\rm{\partial }}Pr({{\rm{\Gamma }}}_{\tau }|{\bf{r}}(0))}{{\rm{\partial }}{\bf{r}}(0)}]{\mathscr{D}}{{\rm{\Gamma }}}_{\tau }\\  & = & -\theta {k}_{{\rm{B}}}\{\int \frac{{\rm{\partial }}Pr({{\rm{\Gamma }}}_{\tau }|{\bf{r}}(0))}{{\rm{\partial }}{\bf{r}}(0)}{\rm{l}}{\rm{n}}[Pr({{\rm{\Gamma }}}_{\tau }|{\bf{r}}(0))]{\mathscr{D}}{{\rm{\Gamma }}}_{\tau }\\  &  & +\mathop{\underbrace{\int \frac{{\rm{\partial }}Pr({{\rm{\Gamma }}}_{\tau }|{\bf{r}}(0))}{{\rm{\partial }}{\bf{r}}(0)}{\mathscr{D}}{{\rm{\Gamma }}}_{\tau }}}\limits_{=0}\}\\  & = & -\theta {k}_{{\rm{B}}}\int \frac{{\rm{\partial }}Pr({{\rm{\Gamma }}}_{\tau }|{\bf{r}}(0))}{{\rm{\partial }}{\bf{r}}(0)}{\rm{l}}{\rm{n}}[Pr({{\rm{\Gamma }}}_{\tau }|{\bf{r}}(0))]{\mathscr{D}}{{\rm{\Gamma }}}_{\tau }.\end{array}$$

We can assume deterministic behavior within one small sub-interval [*t*, *t* + *ε*]. Therefore a conditional path probability can be decomposed into the probabilities of its intervals in the following way:

7 $${\rm{\Pr }}({{\rm{\Gamma }}}_{\tau }|{\bf{r}}\mathrm{(0))}=[\mathop{\prod }\limits_{n\mathrm{=1}}^{N}{\rm{\Pr }}({{\rm{\Gamma }}}_{\varepsilon }|{\bf{r}}({t}_{n}))]{\rm{\Pr }}({{\rm{\Gamma }}}_{\varepsilon }|{\bf{r}}\mathrm{(0)),}$$

where Γ_*ε*_ denotes a path of length *ε* and *τ* = *Nε*. Accordingly, we can express the gradient of the probability as

8 $$\frac{\partial {\rm{\Pr }}({{\rm{\Gamma }}}_{\tau }|{\bf{r}}\mathrm{(0))}}{\partial {\bf{r}}\mathrm{(0)}}=[\mathop{\prod }\limits_{n\mathrm{=1}}^{N}Pr({{\rm{\Gamma }}}_{\varepsilon }|{\bf{r}}({t}_{n}))]\frac{\partial Pr({{\rm{\Gamma }}}_{\varepsilon }|{\bf{r}}\mathrm{(0))}}{\partial {\bf{r}}\mathrm{(0)}}$$

Since Γ_*ε*_ can be seen as the path from **r**(0) to **r**(*ε*) in one step, the gradient in probability of jumping from **r**(0) to **r**(*ε*) with respect to **r**(0) is equal to the negative gradient in probability of jumping from **r**(0) to **r**(*ε*) with respect to **r**(*ε*):

9 $$\frac{\partial Pr({{\rm{\Gamma }}}_{\varepsilon }|{\bf{r}}\mathrm{(0))}}{\partial {\bf{r}}\mathrm{(0)}}=-\frac{\partial Pr({{\rm{\Gamma }}}_{\varepsilon }|{\bf{r}}\mathrm{(0))}}{\partial {\bf{r}}(\varepsilon )}\mathrm{.}$$

By Taylor expanding the position **r**(*ε*) we find

10 $${\bf{r}}(\varepsilon )={\bf{r}}\mathrm{(0)}+\frac{{\bf{p}}\mathrm{(0)}}{2m}\varepsilon +\frac{{\bf{f}}\mathrm{(0)}+{\bf{h}}\mathrm{(0)}}{2m}{\varepsilon }^{2},$$

where **f**(*t*) denotes a random Gaussian force with

11 $$\langle {\bf{f}}(t)\rangle =\mathrm{0,}\,\langle {\bf{f}}(t){\bf{f}}(t^{\prime} )\rangle =\frac{m{k}_{{\rm{B}}}T}{{\varepsilon }^{2}}{\delta }_{ij}\delta (t-t^{\prime} ),$$

and where *δ*(*t*) is the Dirac *δ*-distribution, and *δ*_*ij*_ is the Kronecker delta.

Because these are equilibrium, Brownian trajectories, for very short times the probability distribution of the displacements is Gaussian in **r**(*ε*)

12 $${\rm{\Pr }}({{\rm{\Gamma }}}_{\varepsilon }|{\bf{r}}\mathrm{(0))} \sim exp(-\frac{1}{2}\frac{{({\bf{r}}(\varepsilon )-\langle {\bf{r}}(\varepsilon )\rangle )}^{2}}{\langle {{\bf{r}}}^{2}(\varepsilon )\rangle -{\langle {\bf{r}}(\varepsilon )\rangle }^{2}})$$

13 $$\begin{array}{rcl}\Rightarrow \frac{\partial Pr({{\rm{\Gamma }}}_{\varepsilon }|{\bf{r}}\mathrm{(0))}}{\partial {\bf{r}}(\varepsilon )} & = & -\frac{{\bf{r}}(\varepsilon )-\langle {\bf{r}}(\varepsilon )\rangle }{\langle {{\bf{r}}}^{2}(\varepsilon )\rangle -{\langle {\bf{r}}(\varepsilon )\rangle }^{2}}Pr({{\rm{\Gamma }}}_{\varepsilon }|{\bf{r}}\mathrm{(0))}\\  & = & -\frac{2{\bf{f}}\mathrm{(0)}}{{k}_{{\rm{B}}}T}Pr({{\rm{\Gamma }}}_{\varepsilon }|{\bf{r}}\mathrm{(0))}\,\mathrm{.}\end{array}$$

Using this last result Eq. (9) takes the form

14 $$\frac{\partial {\rm{\Pr }}({{\rm{\Gamma }}}_{\tau }|{\bf{r}}\mathrm{(0))}}{\partial {\bf{r}}\mathrm{(0)}}=[\mathop{\prod }\limits_{n=1}^{N}{\rm{\Pr }}({{\rm{\Gamma }}}_{\varepsilon }|{\bf{r}}({t}_{n}))]\frac{2{\bf{f}}\mathrm{(0)}}{{k}_{{\rm{B}}}T}{\rm{\Pr }}({{\rm{\Gamma }}}_{\varepsilon }|{\bf{r}}\mathrm{(0)).}$$

Plugging this last equation in Eq. (6) we obtain

15 $${\bf{F}}({\bf{r}};\tau )=-\frac{2\theta }{T}\int \,{\bf{f}}\mathrm{(0)}Pr({{\rm{\Gamma }}}_{\tau }|{\bf{r}}\mathrm{(0))}\,\mathrm{ln}\,[{\rm{\Pr }}({{\rm{\Gamma }}}_{\tau }|{\bf{r}}\mathrm{(0))]}{\mathscr{D}}{{\rm{\Gamma }}}_{\tau }\mathrm{.}$$

To estimate the probabilities *Pr*(Γ_*τ*_|**r**(0)) we use *N*_Ω_ Brownian trajectories exploring the available space for a finite time (horizon) *τ*. Every sampling trajectory starts from the current system state **r**(0). We assign a uniform probability for all paths within a neighborhood of a sampled path based on the volume Ω_*n*_ explored ${\rm{\Pr }}({{\rm{\Gamma }}}_{\tau ,n}|{\bf{r}}\mathrm{(0))}{\mathscr{D}}{{\rm{\Omega }}}_{n}^{-1}$. From the normalization condition we find

16 $${{\rm{\Omega }}}_{n}=\frac{1}{{N}_{{\rm{\Omega }}}{\rm{\Pr }}({{\rm{\Gamma }}}_{\tau ,n}|{\bf{r}}\mathrm{(0))}}\mathrm{.}$$

Thus, our estimate of the force in Eq. (15) is

17 $$\begin{array}{ccc}{\bf{F}}({\bf{r}}(0);\tau ) & = & -\frac{\theta }{T}\int {\bf{f}}(0)Pr({{\rm{\Gamma }}}_{\tau }|{\bf{r}}(0))\,{\rm{l}}{\rm{n}}\,[Pr({{\rm{\Gamma }}}_{\tau }|{\bf{r}}(0))]{\mathscr{D}}{{\rm{\Gamma }}}_{\tau }\\  & \approx  & -\frac{2\theta }{T}\langle \mathop{\sum }\limits_{n=1}^{{N}_{{\rm{\Omega }}}}\,{{\bf{f}}}_{n}(0)\frac{1}{{N}_{{\rm{\Omega }}}{{\rm{\Omega }}}_{n}}{\rm{l}}{\rm{n}}(\frac{1}{{N}_{{\rm{\Omega }}}{{\rm{\Omega }}}_{n}}){{\rm{\Omega }}}_{n}\rangle \\  & = & \frac{2\theta }{T{N}_{{\rm{\Omega }}}}\langle \mathop{\sum }\limits_{n=1}^{{N}_{{\rm{\Omega }}}}\,{{\bf{f}}}_{n}(0)\,{\rm{l}}{\rm{n}}({N}_{{\rm{\Omega }}}{{\rm{\Omega }}}_{n})\rangle \\  & = & \frac{2\theta }{T{N}_{{\rm{\Omega }}}}\{\mathop{\underbrace{\langle \mathop{\sum }\limits_{n=1}^{{N}_{{\rm{\Omega }}}}\,{{\bf{f}}}_{n}(0){\rm{l}}{\rm{n}}({N}_{{\rm{\Omega }}})\rangle }}\limits_{=0}+\langle \mathop{\sum }\limits_{n=1}^{{N}_{{\rm{\Omega }}}}\,{{\bf{f}}}_{n}(0)\,{\rm{l}}{\rm{n}}({{\rm{\Omega }}}_{n})\rangle \}\\  & = & \frac{2\theta }{T{N}_{{\rm{\Omega }}}}\langle \mathop{\sum }\limits_{n=1}^{{N}_{{\rm{\Omega }}}}\,{{\bf{f}}}_{n}(0)\,{\rm{l}}{\rm{n}}({{\rm{\Omega }}}_{n})\rangle .\end{array}$$

Finally, by adding a vanishing term, we find

18 $$\begin{array}{ccc}{\bf{F}}({\bf{r}}(0);\tau ) & = & \frac{2\theta }{T{N}_{{\rm{\Omega }}}}\langle \mathop{\sum }\limits_{n=1}^{{N}_{{\rm{\Omega }}}}\{{{\bf{f}}}_{n}(0){\rm{l}}{\rm{n}}({{\rm{\Omega }}}_{n})-{{\bf{f}}}_{n}(0){\rm{l}}{\rm{n}}\langle {{\rm{\Omega }}}_{n}\rangle \}\rangle \\  & = & \frac{2\theta }{T{N}_{{\rm{\Omega }}}}\langle \mathop{\sum }\limits_{n=1}^{{N}_{{\rm{\Omega }}}}\,\{{{\bf{f}}}_{n}(0)(\,{\rm{l}}{\rm{n}}({{\rm{\Omega }}}_{n})-\,{\rm{l}}{\rm{n}}\,\langle {{\rm{\Omega }}}_{n}\rangle )\}\rangle \\  & = & \frac{2\theta }{T{N}_{{\rm{\Omega }}}}\langle \mathop{\sum }\limits_{n=1}^{{N}_{{\rm{\Omega }}}}\,{{\bf{f}}}_{n}(0)\,{\rm{l}}{\rm{n}}(\frac{{{\rm{\Omega }}}_{n}}{\langle {{\rm{\Omega }}}_{{n}^{{\rm{^{\prime} }}}}\rangle })\rangle .\end{array}$$

The volume of every trajectory is defined as ${{\rm{\Omega }}}_{n}^{-1}={N}_{{\rm{\Omega }}}P({{\rm{\Gamma }}}_{\tau }(t)|{{\bf{r}}}_{0})$, and we approximate it through the radius of gyration *R* of all *K* positions of the sampling trajectory relative to their mean position ${{\rm{\Omega }}}_{n}\approx {R}^{2}=\frac{1}{K}{\sum }_{k\mathrm{=1}}^{K}\,{({{\bf{r}}}_{k}-\bar{{\bf{r}}})}^{2}$.

### Measure of anisotropy

The Minkowski tensor ${{\bf{W}}}_{2}^{\mathrm{1,1}}(C)\equiv \frac{1}{2}{\int }_{\partial C}\,{\bf{r}}\odot {\bf{n}}\,{G}_{2}\,dr$ provides a measure of anisotropic morphologies^34,35^. It is a second rank symmetric tensor, where *G*_2_ = (*κ*_1_ + *κ*_2_)/2 is the local curvature, **r** the position vector, **n** the normal vector to the surface ∂*C* of a body, and ${(a\odot b)}_{ij}\equiv ({a}_{i}{b}_{j}+{a}_{j}{b}_{i})/2$ is the symmetric tensor product of vectors **a** and **b**. The anisotropy parameter $\alpha \equiv \frac{{\beta }_{1}-{\beta }_{2}}{{\beta }_{1}+{\beta }_{2}}$, where *β*_1_ and *β*_2_ are the largest and smallest eigenvalues of ${{\bf{W}}}_{2}^{\mathrm{1,1}}$, respectively, gives a measure of anisotropy of the pattern.
